# Malformations in a cohort of 284 women with Mayer-Rokitansky-Küster-Hauser syndrome (MRKH)

**DOI:** 10.1186/1477-7827-10-57

**Published:** 2012-08-20

**Authors:** Patricia G Oppelt, Johannes Lermann, Reiner Strick, Ralf Dittrich, Pamela Strissel, Ingo Rettig, Christine Schulze, Stefan P Renner, Matthias W Beckmann, Sara Brucker, Katharina Rall, Andreas Mueller

**Affiliations:** 1 Department of Obstetrics and Gynecology, Erlangen University Hospital, Universitätsstrasse 21-23, Erlangen, 91054, Germany; 2 Department of Internal Medicine IV, Tübingen University Hospital, Otfried-Müller-Straße 10, Tübingen, 72076, Germany; 3 Department of Obstetrics and Gynecology, Tübingen University Hospital, Calwerstr. 7, Tübingen, 72076, Germany

**Keywords:** MRKH syndrome, Diagnosis, Staging, Malformations, VCUAM, Renal abnormalities

## Abstract

**Background:**

The aim of this retrospective study was to describe the spectrum of genital and associated malformations in women with Mayer-Rokitansky-Küster-Hauser syndrome using evaluated diagnostic procedures and the Vagina Cervix Uterus Adnex – associated Malformation classification system (VCUAM).

**Methods:**

290 women with MRKH syndrome were clinically evaluated with using clinical examinations, abdominal and perineal/rectal ultrasound, MRI, and laparoscopy.

**Results:**

Classification of female genital malformation according to the Vagina Cervix Uterus Adnex – associated Malformation classification system was possible in 284 women (97.9%). Complete atresia of Vagina (V5b) and bilateral atresia of Cervix (C2b) were found in 284 patients (100%). Uterus: bilateral rudimentary or a plastic uterine horns were found in 239 women (84.2%). Adnexa: normal Adnexa were found in 248 women (87.3%). Malformations: associated malformations were found in 126 of 282 evaluable women (44.7%), 84 women (29.6%) had malformations of the renal system. Of 284 women with Mayer-Rokitansky-Küster-Hauser syndrome 212 women (74.7%) could be classified as V5bC2bU4bA0. The most frequent classification was V5bC2bU4bA0M0 (46.8%) diagnosed in 133 of 284 women.

**Conclusions:**

Complete atresia of vagina and cervix were found in all patients, variable malformations were found with uterus and adnexa. A variety of associated malformations were present, predominantly of the renal system. It is therefore recommended that all patients with genital malformations should be evaluated for renal abnormalities.

## Background

The Mayer–Rokitansky–Küster–Hauser (MRKH) syndrome describes women with normal female external development and internally with normally regressed mesonephric (Wolffian) ducts, but abnormally absent paramesonephric (Müllerian) ducts. MRKH syndrome involves congenital aplasia of the uterus, cervix and upper two-thirds of the vagina. The syndrome is only revealed when primary amenorrhea is noticed or attempts at coitus are in vain. It occurs as a purely genital malformation (type 1), but also with associated malformations (type 2 and MURCS association; *Mü*llerian *r*enal, *c*ervicothoracic *s*omite abnormalities) [1]. Malformations of the kidneys and urinary tract the skeleton, and more rarely of the heart and central nervous system have been described [2]. The incidence of the syndrome is one in 4,500 female newborns [1]. A failure of fusion of Müllerian duct derivatives during gestational weeks 4–12 results in malformation of the vagina and uterus [3]. However, the precise pathogenetic mechanism is still unknown. MRKH patients have a correctly timed pubarche and thelarche and have a normal female karyotype (46, XX) [3]. The syndrome was first described by Mayer [4] in 1829, and Rokitansky [5] later published a case report on similar malformations. In 1910, Küster wrote the first review on the syndrome [6]. Hauser and Schreiner were the first in 1961 to call it Mayer–Küster–Rokitansky syndrome [7]. The final addition of Hauser to the name resulted in today’s term, Mayer–Rokitansky–Küster–Hauser syndrome.

The *V*agina, *C*ervix, *U*terus, *A*dnex – associated *M*alformation (VCUAM) classification (Table 1) was introduced in 2005 to allow an accurate description of genital and associated malformations [8-11]. 

**Table 1:** **VCUAM classification**[8]

**Organ**	**Stage**	**Description**
Vagina (V)	0	Normal
	1a	Partial hymenal atresia
	1b	Complete hymenal atresia
	2a	Incomplete septate vagina < 50%
	2b	Complete septate vagina
	3	Stenosis of the introitus
	4	Hypoplasia
	5a	Unilateral atresia
	5b	Complete atresia
	S1	Sinus urogenitalis (deep confluence)
	S2	Sinus urogenitalis (middle confluence)
	S3	Sinus urogenitalis (high confluence)
	C	Cloacae
	+	Other
	#	Unknown
Cervix (C)	0	Normal
	1	Duplex cervix
	2a	Unilateral atresia/aplasia
	2b	Bilateral atresia/aplasia
	+	Other
	#	Unknown
Uterus (U)	0	Normal
	1a	Arcuate
	1b	Septate <50% of the uterine cavity
	1c	Septate >50% of the uterine cavity
	2	Bicornate
	3	Hypoplastic uterus
	4a	Unilaterally rudimentary or aplastic
	4b	Bilaterally rudimentary or aplastic
	+	Other
	#	Unknown
Adnexa (A)	0	Normal
	1a	Unilateral tubal malformation, ovaries normal
	1b	Bilateral tubal malformation, ovaries normal
	2a	Unilateral hypoplasia/gonadal streak (including tubal malformation if appropriate)
	2b	Bilateral hypoplasia/gonadal streak (including tubal malformation if appropriate)
	3a	Unilateral aplasia
	3b	Bilateral aplasia
	+	Other
	#	Unknown
Associated malformations (M)	0	None
	R	Renal system
	S	Skeleton
	C	Cardiac
	N	Neurologic
	+	Other
	#	Unknown

Clinical examination, ultrasound, magnetic resonance imaging (MRI), and laparoscopy can help to diagnose MRKH syndrome [9,12-16]. We recently presented a comparison of different diagnostic procedures for the correct staging of the malformations according to the VCUAM classification with defined reference methods for the different organs involved in the syndrome. The reference methods were: vagina — clinical examination; cervix/uterus and adnexa — laparoscopy; urinary tract malformations — MRI [17]. The quality of other diagnostic procedures for each organ was expressed as agreement with the reference methods, which was presented as kappa value (*k*). For vagina and cervix only clinical examination was found to be sufficient , for uterus MRI (*k* 0.93) or ultrasound (k 0.83) were found to be sufficient, for adnexa only laparoscopy was found to be sufficient and for urinary tract malformations ultrasound was also found to be sufficient (*k* 0.87) [17].

The aim of this study was to describe the spectrum of malformations in a large cohort of 290 women with MRKH syndrome in order to verify the most common subtypes of this syndrome.

## Methods

The study is a two centre retrospective analysis of 290 women who were treated between January 2000 and October 2011 for MRKH syndrome in the University hospitals of Erlangen and Tübingen (Germany).

All patient files were systematically analysed and all data were used anonymously. Each malformation of the organs was classified in accordance with the VCUAM classification [8,9], based upon the results of the reference methods, for vagina — clinical examination, for cervix and adnexa — laparoscopy for uterus – ultrasound and for associated malformations — MRI. In case a classification for each organ was not possible using the reference methods other diagnostic procedures with a good agreement with the reference methods (*k* > 0.80) were considered. *Vagina:* all women received a regular gynecological examination, including rectal palpation. *Uterus:* ultrasound examinations were performed transabdominally using standard 2–7 MHz probes, or perineally/transrectally using standard 3.3–10 MHz probes in all women. *Cervix and adnexa:* all women underwent laparoscopy, sometimes as part of a modified laparoscopic Vecchietti operation [18-20]. *Malformations:* Ultrasound examinations were performed transabdominally using standard 2–7 MHz probes and/or MRI was carried out with a 1.5-T magnetic resonance system in some women. Approval for conducting basic research and compiling the relevant documentation to perform this study was received through Institution review board (IRB) approval No. 3074.

### Statistical analysis

All data are presented as frequencies and percentages using Microsoft Office Excel 2009 and IBM SPSS Statistics 19.

## Results

The patient files of 290 women with MRKH syndrome who were diagnosed and treated during the study period were analysed and 284 files were eligible for inclusion in the analysis. Six patient files were excluded because of missing examination results. All 284 women underwent clinical examination, ultrasound and laparoscopy. A diagnostic laparoscopy and/or therapeutic intervention were performed with 275 women (96.8%). MRI of the urinary tract was carried out in most women; in addition an MRI was performed for some women to investigate skeletal malformations. Women with skeletal, neuronal or cardiac malformations were further examined, using thorax X-ray, echocardiography, audiometry or visual tests. The mean age of the women at the time of inclusion was 26.2 (SD 8.8) years and the mean body mass index at the time of diagnostic or therapeutic intervention was 23.1 (SD 4.8) kg/m^2^.

### VCUAM classifications

#### Vagina (V)

284 women (100%) showed stage V5b (complete atresia) of the vagina.

#### Cervix (C)

284 women (100%) showed stage 2b (bilateral atresia/aplasia).

#### Uterus (U)

239 women (84.2%) showed stage 4b (bilateral rudimentary or aplastic), 27 women (9.5%) showed stage 4a (unilateral rudimentary or aplastic), eight women (2.8%) showed other and eight women (2.8%) were not classifiable. Two women (0.7%) showed stage 3 (hypoplastic uterus).

#### Adnexa (A)

248 women (87.3%) showed stage 0 (normal adnexa), nine (3.2%) were not classifiable, ten (3.5%) showed stage 2a (unilateral hypoplasia), seven (2.5%) showed 1b (bilateral tubal malformation) and six women (2.1%) showed stage 3a (unilateral aplasia). Two women (0.7%) showed stage 2b (bilateral hypoplasia/gonadal streak) and one women (0.4%) stage 1a (unilateral tubal malformation). One woman (0.4%) showed other malformations.

#### Malformations (M)

In 156 women (54.9%) of 284 women no associated malformations were diagnosed (stage 0). In 53 women (18.7%) malformations of the renal system (stage R) and in 22 women (7.7%) malformations of the skeleton (stage S) were found. Two women (0.7%) were not classifiable, five (1.8%) showed other malformations, two women (0.7%) showed cardiac malformations and four woman (1.4%) showed neurologic malformations. Moreover in 38 women (13.4%) different combinations (renal, skeleton, cardiac, neurologic, others) were found. Table 2 illustrates associated malformations of 282 women, excluding two women which were not classifiable. Eighty-four patients (29.6%) showed renal malformations where two women were excluded because of an unknown renal status (see Table 3). Fifty-three of these 84 patients (64.4%) or 18.8% of all patients with known malformations showed a renal agenesis.

**Table 2:** VCUAM classification of 284 patients with MRKH syndrome

**Vagina**	**n (%)**	**Cervix**	**n (%)**	**Uterus**	**n (%)**	**Adnexa**	**n (%)**	**Malformation**	**n (%)**
V5b	284 (100%)	C2b	284 (100%)	U0	0 (0%)	A0	248 (87.3%)	M0	156 (54.9%)
				U1a	0 (0%)	A1a	1 (0.4%)	MR	53 (18.7%)
				U1b	0 (0%)	A1b	7 (2.5%)	MS	22 (7.7%)
				U1c	0 (0%)	A2a	10 (3.5%)	MC	2 (0.7%)
				U2	0 (0%)	A2b	2 (0.7%)	MN	4 (1.4%)
				U3	2 (0.7%)	A3a	6 (2.1%)	M+	5 (1.8%)
				U4a	27 (9.5%)	A3b	0 (0%)	M#	2 (0.7%)
				U4b	239 (84.2%)	A+	1 (0.4%)	Combinations	
				U+	8 (2.8%)	A#	9 (3.2%)	MR+	3 (1.1%)
				U#	8 (2.8%)			MRC	1 (0.4%)
								MRC+	1 (0.4)
								MRN	2 (0.7%)
								MRS	15 (5.3)
								MRS+	4 (1.4%)
								MRSC	2 (0.7)
								MRSN	3 (1.1%)
								MS+	2 (0.7%)
								MSC	1 (0.4)
								MSCN	2 (0.7%)
								MSN	3 (1.1)
								MC+	1 (0.4)

**Table 3:** Specification of 82 renal malformations diagnosed in 284 patients with MRKH syndrome

**Renal malformations**	**patients**
ureter malformation	2
renal malrotation	2
pelvic kidney	8
pelvic kidneys	1
pelvic kidney + ureter malformation	1
double renal pelvis	1
duplex kidney	5
duplex kidneys	1
duplex kidney + ureter malformation	1
cirrhosis of the kidney	3
horseshoe kidney	3
horseshoe kidney + ureter malformation + cystic kidney disease	1
kidney agenesis	35
kidney agenesis + duplex ureter	1
kidney agenesis + pelvic kidney	15
kidney agenesis + pelvic kidney + renal malrotation	1
kidney agenesis + bladder malformation + persistent urachus	1

**Figure 1:**
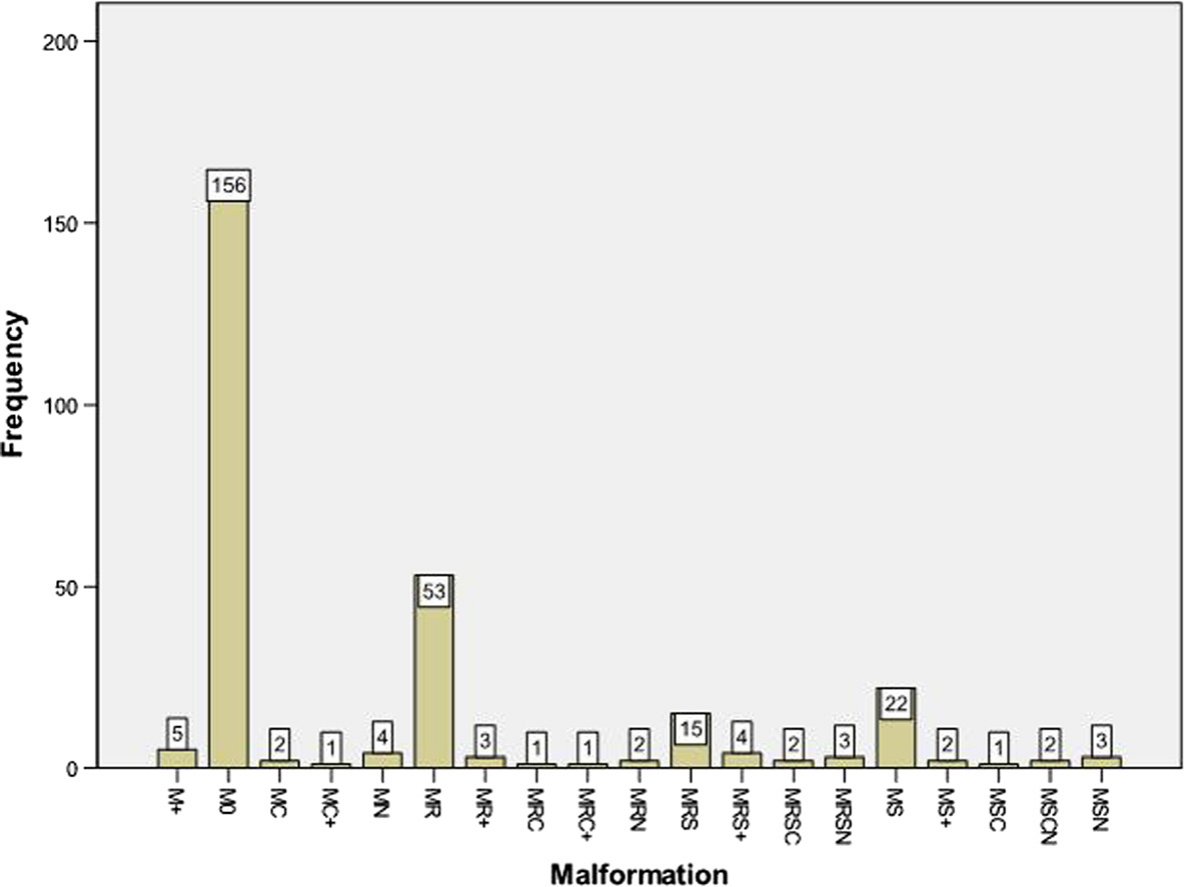
**Malformations of 282 patients with MRKH syndrom excluding M#.** M + = 5, M0 = 156, MC = 2, MC + = 1, MN = 4, MR = 53, MR + = 3, MRC = 1, MRC + = 1, MRN = 2, MRS = 15, MRS + = 4, MRSC = 2, MRSN = 3, MS = 22, MS + = 2, MSC = 1, MSCN = 2; MSN = 3.

In summary, excluding associated malformations from 284 women with MRKH syndrome, 212 women could be classified as complete atresia of the vagina (V5b), bilateral atresia of the cervix (C2b), bilateral aplastic uterus (U4b) and normal adnexa (A0) (74.7%). The most frequent VCUAM classification was V5bC2bU4bA0M0 diagnosed in 133 (46.8%) of 284 women. The spectrum of associated malformations is shown in Figure 1.

## Discussion

To the best of our knowledge, this study represents the largest cohort of 284 women with MRKH syndrome diagnosed with evaluated and standardized diagnostic procedures and grouped according to a defined classification system. There is a unique occurrence in malformations of the distal genital system; vagina, cervix and uterus. The spectrum of variations accumulates in the more proximal part of the female genital system with a variety of adnexal and associated malformations. Most associated malformations were found in the renal system along with additional numerous combinations of different malformations (see Figure 1).

Examination of women with MRKH syndrome revealed according to the definition of MRKH syndrome an absence or severe hypoplasia of the upper vagina as well as uterine agenesis. In the present study 100% of MRKH women showed a complete atresia of vagina, although 1–3 cm of the lower vagina can be present. We found in 100% of patients an aplasia of the cervix (Table 2). In our study 87.3% of patients showed normal adnexa (stage A0), ten (3.5%) showed stage 2a (unilateral hypoplasia), seven (2.5%) showed 1b (bilateral tubal malformation) and six women (2.1%) showed stage 3a (unilateral aplasia). Two women (0.7%) showed stage 2b (bilateral hypoplasia/gonadal streak) and one women (0.4%) stage 1a (unilateral tubal malformation). One woman (0.4%) showed other malformations (Table 2). We did not find a tendency toward polycystic ovaries as some authors reported [7,21,22]. We agree with Rokitansky, Bompiani and Oppelt et al. who described hypoplastic or aplastic ovaries only in a few cases [3,5,23]. Characteristic for all MRKH patients is the unilateral or bilateral hypoplasia of the uterus. In our study 84.2% showed bilaterally rudimentary or aplastic uterus, in 9.5% a unilaterally rudimentary or aplastic uterus was diagnosed. Two (0.7%) women had a hypoplastic uterus (Table 2). These proportions of the uterus developmental state in MRKH patients was similar to Guerrier et al. [24].

The present analysis showed that 44.4% of the patients were affected by associated malformations. Excluding women which were not classifiable (M#) (0.7%), we found associated malformations in 44.7% of patients (Table 3). The rate of associated malformations in patients with MRKH syndrome reported in the literature was between 53% [3] and 64% [25]. Although MRKH patients have a normal female karyotype, in case of severe hormonal alterations, like the androgen insensitivity syndrome, it is recommended that patients should be analysed for chromosomal changes. In our study we focused more on the examination of the renal system, where every woman received an examination by laparoscopy and ultrasound and/or MRI. No direct symptoms of skeletal malformations like dorsal pain, scoliosis etc. excluded MRI analysis. An open inguinal canal was often found during laparoscopy, but only women with an operated inguinal hernia were classified as M+. Malformations of the renal system were seen in up to 32% of the patients and represented the largest proportion of affected organs [3]. 29.6% of our cohort (284 patients) had renal malformations, confirming this as the most frequent associated malformation with MRKH (Table 3). The most frequent renal malformation was a renal agenesis (64.4%).

Furthermore, we support that the associated renal malformations with MRKH can be explained due to the close link between genital and urinary embryonal development.

The human genital tracts are undifferentiated until the 8th week of gestation and are referred to as “bipotential or indifferent” gonads. At this time both the male and female embryo have two symmetrical paired genital ducts: the mesonephric (Wolffian) and the paramesonephric (Müllerian) ducts, which originate from the intermediate mesoderm. Together with the urogenital sinus they provide the bases for internal and external genital development. Only one of the two ductal systems will normally develop further, depending on whether differentiation of a testis or ovary has begun. The distal mesonephric duct is the starting point for a pair of ureteric buds, which grow into the cloaca and induces the overlying metanephros to develop into the primitive kidneys. The ureteric buds and distal portions of the mesonephric ducts are later incorporated into the wall of the primitive bladder to develop into ureters, trigone and bladder neck. In the female embryo, the mesonephric duct regresses completely and the paramesonephric (Müllerian) duct develops into the fallopian tubes, uterus, cervix and upper part of the vagina. Importantly, ovaries originate within the primitive ectoderm, thus are independent of the mesonephros. In the male embryo, testosterone and androstenedione stimulates mesonephric duct development to form the epididymi, vasa deferentia and seminal vesicles, while the Müllerian duct regresses in response to anti-Müllerian-hormone, which is secreted from the Sertoli cells. The disappearance of the Müllerian ducts in the male fetus is completed by 9 to 10 weeks of gestation [26-28].

It is interesting to note that in the general population, urinary tract defects occur in as many as 1:100 live births and constitute the most frequent cause of chronic kidney disease in children [29]. Considering the incidence of MRKH in ~1:4,500 live female births, congenital renal malformations (as well as unilateral renal agenesis) in MRKH patients are higher compared to the general population. This is not surprising due to the association and interaction of the two ductal systems for normal genital and renal development. Combined urogenital malformations are common with estimations of 10 in 100 cases and account for over 30% of all congenital malformations [30]. Malformations of the genital and renal axis are common, e.g. 35% of females where unilateral renal agenesis showed partial or complete duplication of the genital tract [31]; renal agenesis was present in 43% of patients with uterus didelphys and 10% of patients with other genital tract abnormalities had an abnormal or ectopic kidney [32]. As a matter of course all patients with genital malformations should be evaluated for renal abnormalities, may be patients with renal abnormalities should also be assessed for genital malformations.

## Conclusions

There is a unique occurrence in malformations of the distal genital system; vagina, cervix and uterus, while the spectrum of variations accumulates in the more proximal part of the female genital system with a variety of adnexal and associated malformations. Those malformations can be classified precisely using evaluated diagnostic procedures and a standardized classification system. It is recommended that all patients with genital malformations should be evaluated for renal abnormalities, but importantly patients with renal abnormalities should also be assessed for genital malformations.

## Abbreviations

A: Adnexa; C: Cervix; IBM: International Business Machines Corporation; IRB: Institution review board; M: Malformations; MRI: Magnetic resonance imaging; MRKH: Mayer-Rokitansky-Küster-Hauser syndrome; MURCS: Müllerian hypoplasia/aplasia, renal agenesis and cervicothoracic somite dysplasia; N: Neurologic; U: Uterus; V: Vagina; R: Renal; S: Skeleton; SD: Standard deviation; SPSS: Statistical Package for the Social,Sciences.

## Misc

Patricia G Oppelt, Johannes Lermann, Katharina Rall and Andreas Mueller are authors contributed equally to the authorship

## Competing interests

All of the authors declare that they have no competing interest.

## Authors’ contributions

PGO, JL and AM participated in the design of the study and drafted the manuscript. PS, IR, CS, SPR and KR performed the data collection. RS, RD, MWB and SB controlled the classification of the malformations. RS and RD were responsible for statistical analysis and presentation of the data. JL and AM were responsible for the finalizing the manuscript. All authors read and approved the final manuscript.

## Author details

^1^Department of Obstetrics and Gynecology, Erlangen University Hospital, Universitätsstrasse 21-23, Erlangen, 91054, Germany. ^2^Department of Internal Medicine IV, Tübingen University Hospital, Otfried-Müller-Straße 10, Tübingen, 72076, Germany. ^3^Department of Obstetrics and Gynecology, Tübingen University Hospital, Calwerstr. 7, Tübingen, 72076, Germany.
